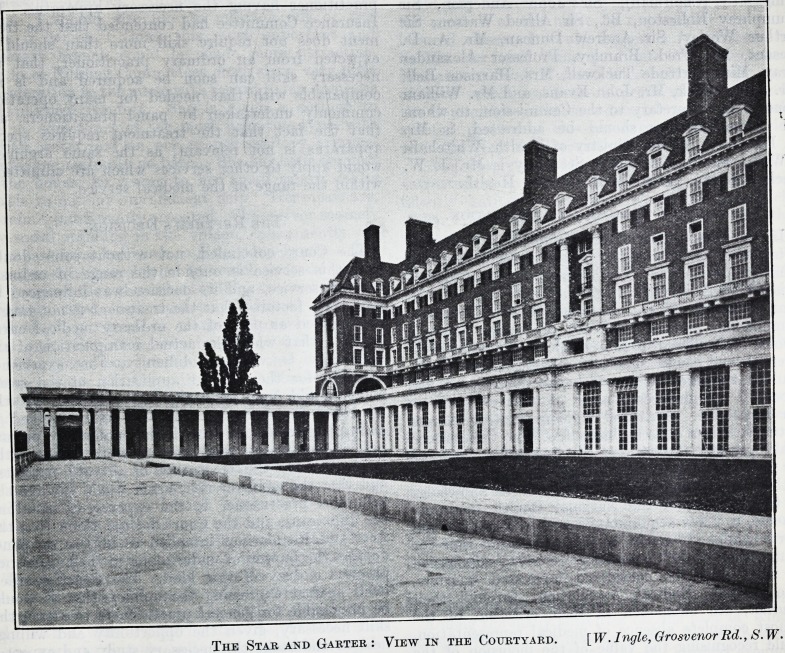# A Haven of Peace

**Published:** 1924-08

**Authors:** 


### A HAVEN OF PEACE.

The noble Star and Garter Home, on Richmond Hill, designed by Sir Edwin Cooper to take the place of the Hotel of that name as a refuge for men hopelessly injured in the war, was visited by the King and Queen on July 10. Her Majesty, who is the Patron, received the deeds of the building as the Women's Memorial of the War, the contributions of women of the Empire having been collected through the agency of the British Women's Hospital Committee. In the course of his speech the King said: " It is singularly happy that the object of a women's War Memorial should be to alleviate the sufferings and to brighten the lives of these men who have sacrificed so much in the cause of their country. As we honour the memory of those who laid down their lives in our defence, so do we testify our gratitude to the many who, in similar circumstances, have incurred bodily disabilities, by assuring them of such relief and comfort as this Home will provide. We deeply sympathise with these noble and humane endeavours, and pray that God's blessing may rest upon the Star and Garter Home."

The Home is of old English red brick and Portland stone, and when mellowed will be a very attractive building indeed. It stands magnificently, and everywhere is an effect of space and light. A Doric colonnade extends the whole length of the south elevation on the lower ground level flanked at each end by a loggia, and on the south, east and west are new terraces with beautiful prospects over the river and the Surrey hills. The building, indeed, commands the whole of the famous view from Richmond Hill. There will be beds for over one hundred and eighty patients, and the Home will provide accommodation not only for victims of the war, but for sailors and soldiers who may be disabled by disease. As Sir Arthur Stanley said to the King and Queen, the Star and Garter promises to become " a haven of peace and comfort to those who deserve the best that we can give."

THE STAR AND GARTER: VIEW IN THE COURTYARD. [*W. Ingle, Grosvenor Rd., S.W.*

### OVERGROWN COTTAGE HOSPITALS.

The Ulverston Cottage Hospital, Lancashire, which was founded in 1873 and enlarged in 1905, is now recorded to be at " the end of its improvements," and a scheme is being started to provide a new hospital in its stead. With twenty-two beds, and inadequate accommodation both for patients and staff, the institution has reached the familiar stage when a progressive cottage hospital has begun to outgrow that designation. Not only is a larger hospital needed, but one which shall be planned to admit future extensions. Mr. C. W. Dean, the chairman of the committee, and, indeed, the committee itself, are alive to the necessity, and it is to be hoped that the subscribers and friends of the hospital will give them the necessary backing.

## Figures and Tables

**Figure f1:**